# Supplementary material for: Structure of the bacterial flagellar hook cap provides insights into a hook assembly mechanism
Source: Commun Biol. 2021 Nov 16;4:1291. doi: 10.1038/s42003-021-02796-6 (PMC8595650; doi:10.1038/s42003-021-02796-6)
Supplement: Supplementary file 2 — Supplementary Information [file 42003_2021_2796_MOESM2_ESM.pdf]

Supplementary Information

Structure of the bacterial flagellar hook cap provides insights into a hook assembly mechanism

Hideyuki Matsunami, Young-Ho Yoon, Katsumi Imada, Keiichi Namba and Fadel A. Samatey

This file contains Supplementary Figures 1–8, Supplementary Tables 1–4, and Supplementary References.

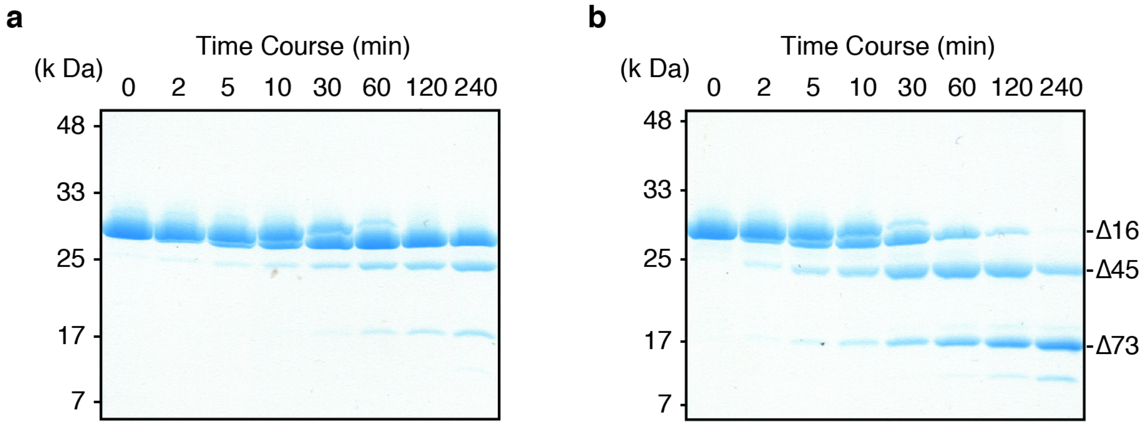

Supplementary Figure 1: Limited proteolysis of FlgD. **a**,**b**, Purified FlgD pentamer (**a**) and monomer (**b**) were incubated with trypsin at a ratio of 300:1 (w/w) in 20 mM Tris-HCl (pH 8.0) at 25°C. The three major digested fragments were analyzed by Time-of-flight mass spectroscopy. The positions of digestion were identified to be the C-termini of Lys16 (Δ16), Lys45 (Δ45) and Lys73 (Δ73).

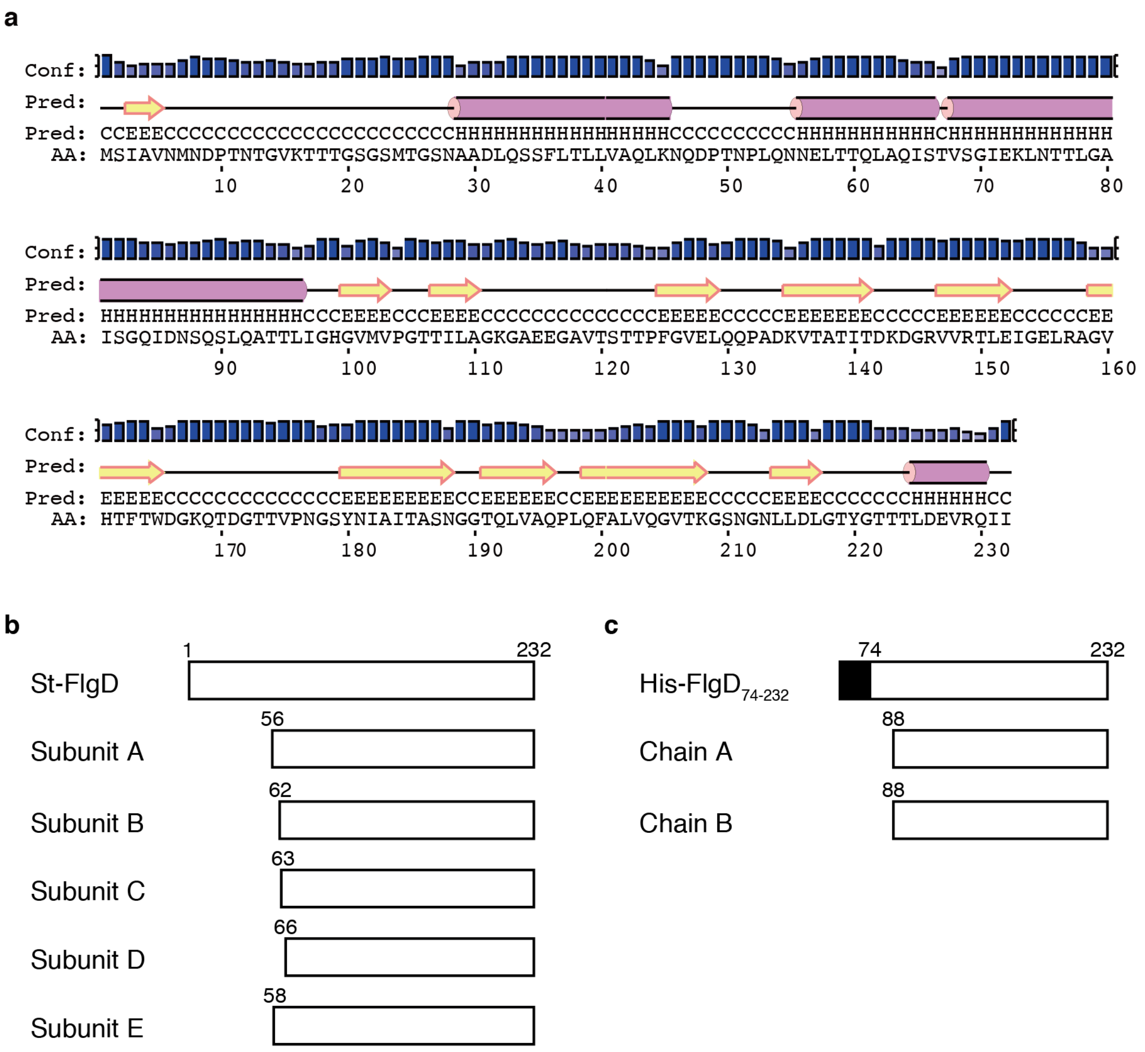

Supplementary Figure 2: **Secondary structure prediction of FlgD and schematic representation of the sequence regions of FlgD for which the structures were determined in this study**. **a**, Secondary structures of *S. enterica* FlgD calculated by *PSIpred*. In addition to the confidence levels of prediction, characters "C", "E" and "H" denote coil, β-sheet and α-helix, respectively. **b**, In the hook cap model, each of the N-terminal part is partly missing. **c**, The two FlgD fragments in the asymmetric unit were modeled for the construct $FlgD_{74\text{-}232}$.

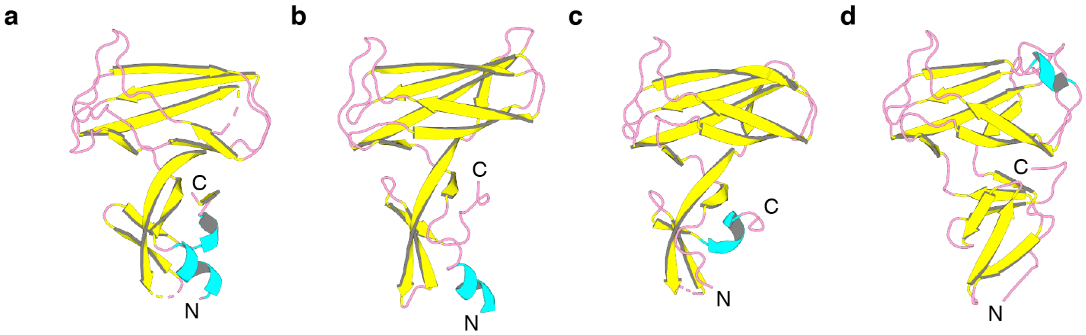

e

```
6IEF_chainA    1   NSQSLQATTLIGHGVMVPGTTILAGKGAEEGAVTSTTPFGVELQQPA----DKVTATITDKDGRVVRTLE   66
3c12_chainA    1   DQVLK-GAALVGHNVLVPSAQVAIDA-------TGSAKGVVAA-TSA----GFVNFEITDANGTFVKQLS   57
3osv_chainA    1   -------SSLVGRKVIVATDKSVVDT-------KDTFKASLNLPVSS----SNVWVNVYDDKGTVVNRIN   52
4zzk_chainA    1   N-----SVSMIGKIAETDVSGANFDG-------NNKLSFSLFFDEKIDASKGVPAIQILNENNELVKTIP   58
Consensus_ss:       hhhh hhhh   eeeee  eeee           eeeeeeee         eeeeeee     eeeeee

6IEF_chainA   67   IG--ELRAGVHTFTWDGKQTDGTTVPNGSYNIAITASN---GGTQLVAQPLQFALVQGVTKGSNGNLLDL  131
3c12_chainA   58   VP--ASAAGEVSFAWDGTDANGNRMAAGKYGITATQTDT--AGAKSKLATYVDAPVDSVTIGSDGLYLNL  123
3osv_chainA   53   LG--QQAAGSVSFMWDGKDSSGNIMPPGTYKFEAQTSI---DGKTYGLQTYLPANVDSVTLG-QELMLNL  116
4zzk_chainA   59   LKDYNGQKGYINFEWDGTNEKGEKVPKGNYKIKAEYNLDSHSKQYLQTR-IGRGEVESVIFDKGKPMLRM  127
Consensus_ss:               eeeeee              eeeeeeeee     eeeeeeeeeeeeeeeee     eeeee

6IEF_chainA  132   GTYGTTTLDEVRQII-----  146
3c12_chainA  124   TGLGTSPLANVLRVS-----  138
3osv_chainA  117   AGLGSIALSKVQIIG-----  131
4zzk_chainA  128   GE-MILPIDSAIEFYKPDQK  146
Consensus_ss:      e   eeehhheeee
```

Supplementary Figure 3: Structural comparison of FlgD proteins. a–d, Crystal structures of $FlgD_{74\text{-}232}$ from *S. enterica* (PDB-id: 6IEF; chain A) (**a**), *X. campestris* (PDB-id: 3C12; chain A) (**b**), *P. aeruginosa* (PDB-id: 3OSV; chain A) (**c**) and *H. pylori* (PDB-id: 4ZZK, chain A) (**d**), shown in Cα ribbon representation with *PyMOL*. The secondary structure elements of α-helices and β-sheets are colored cyan and yellow, respectively. (**e**) Structure-based multiple sequence alignment of FlgD models. Amino-acid sequences of FlgD models deposited in PDB were aligned with *PROMALS3D* server[1]. Consensus secondary structures are shown as "h" (α-helix) and "e" (β-sheet) at the bottom and highly conserved residues are colored red.

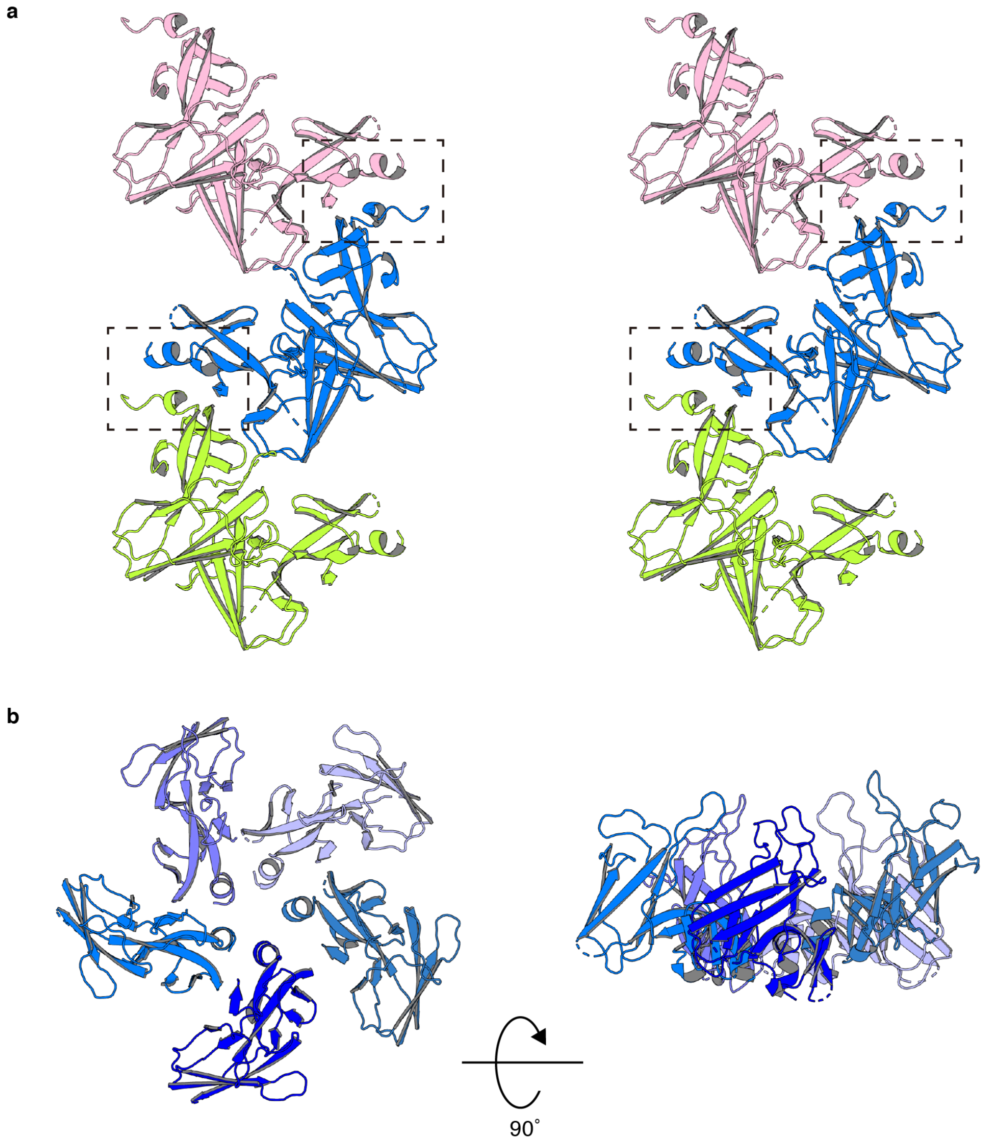

Supplementary Figure 4: Crystal packing of FlgD$_{74\text{-}232}$ and a pentamer cap model made by using one of the crystal contact. a, A stereo view of the three FlgD$_{74\text{-}232}$ dimers in the crystal along the crystallographic *a* axis. Each dimers in the asymmetric unit are colored in light pink, marine and lime, respectively. Crystal contacts of each dimer were boxed with dashed line. **b**, Top and side views of the pentamer model produced by the FlgD$_{74\text{-}232}$ crystal structures by using one of the crystal packing interactions indicated in (**a**). Two FlgD$_{74\text{-}232}$ molecules in the neighboring asymmetric unit were extracted and aligned to produce the pentamer.

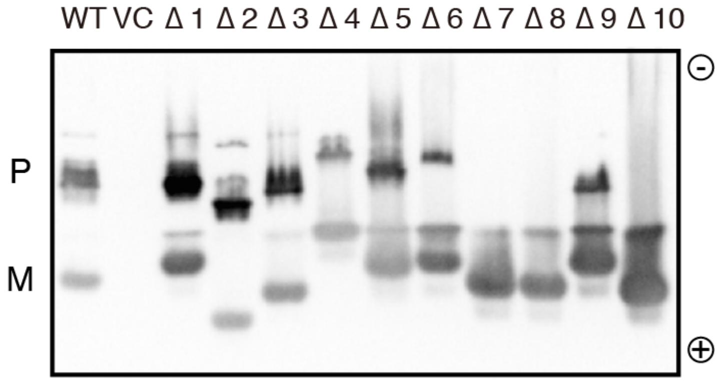

Supplementary Figure 5: Pentamer formation of the *S. enterica* FlgD deletion variants *in vivo*. FlgD variants expressed in *E. coli* BL21(DE3) were detected by Immunoblotting after separation by Native PAGE under non-denatured condition. The migrated bands corresponding to the pentamer and the monomer are indicated by “P” and “M” on the left, respectively. Proteins migrated toward to the positive electrode (+) from negative (-).

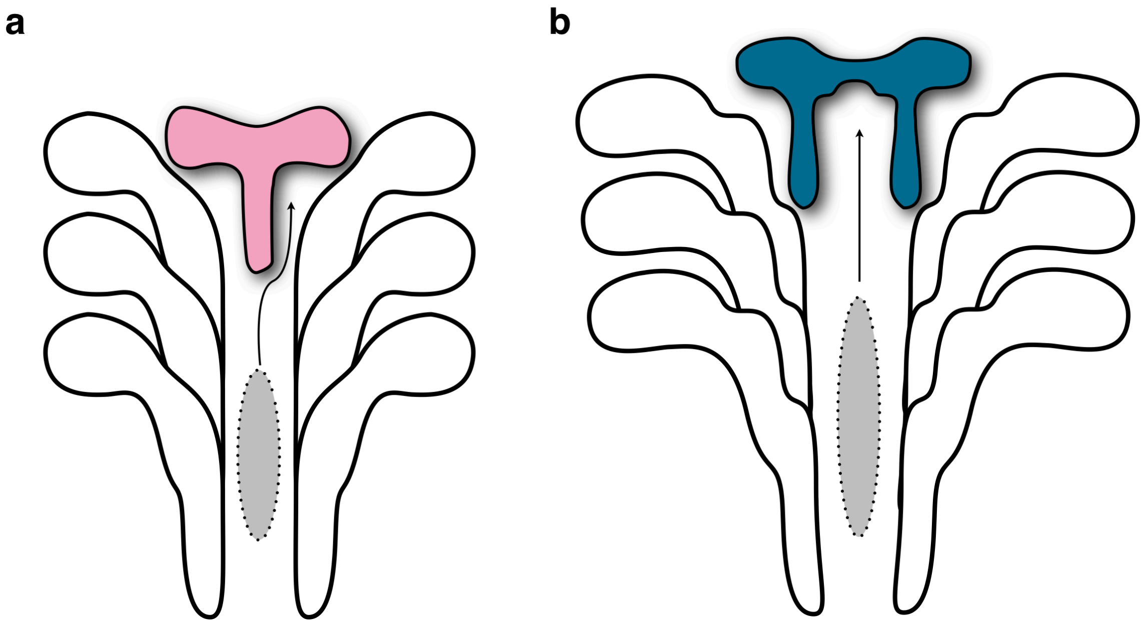

Supplementary Figure 6: Schematic drawings of the subunit folding and assembly for the hook and the filament. The hook cap (pink) bound to the growing tip of the hook (**a**) and the filament cap (blue) at the tip of the filament (**b**). Exported proteins inside the central channel are shown in gray. Folding of the hook protein takes place between the hook cap and the distal end of the growing hook, while the filament protein folds within the folding chamber of the filament cap.

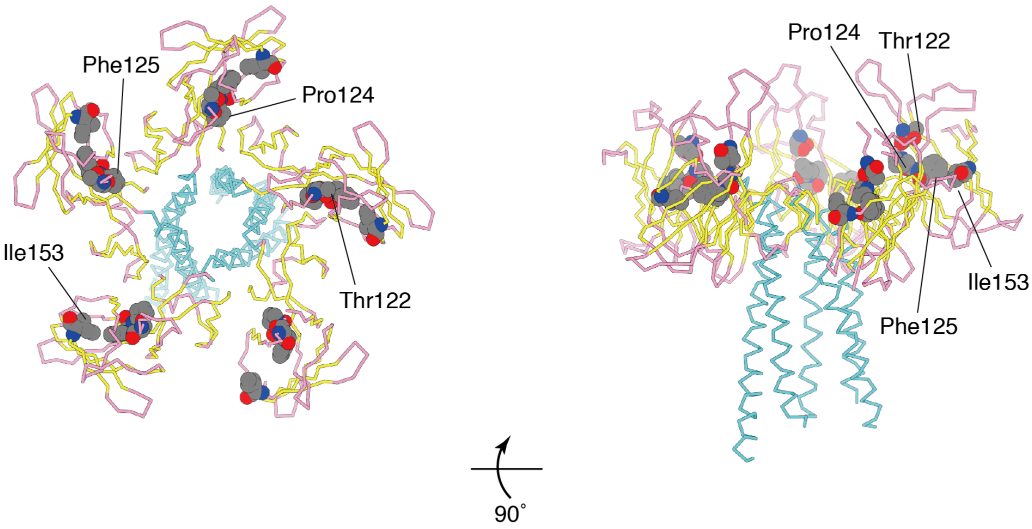

Supplementary Figure 7: Mutated residues in pseudorevertants mapped on the hook cap model. The five subunits of the hook cap are shown in Cα stick representation with colors representing secondary-structure elements (α-helices in cyan, β-sheets in yellow, and loops in pink) and the side chain atoms of mutated residues are shown in spheres with colors for elements: carbon in gray, nitrogen in blue and oxygen in red. Mutated residues shown are Thr 122, Pro 124, Phe 125 and Ile 153.

c

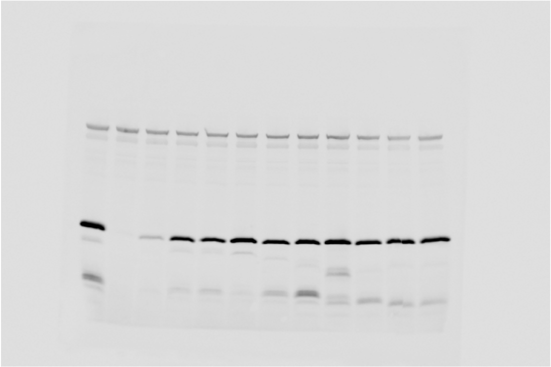

e

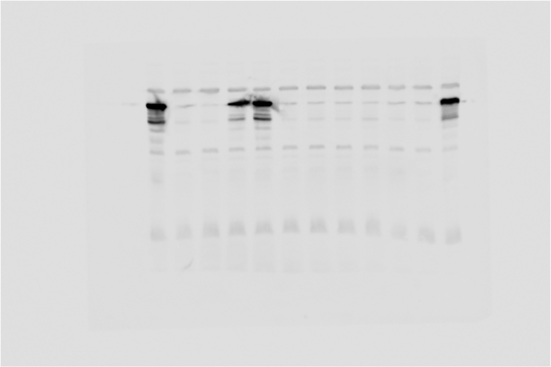

d

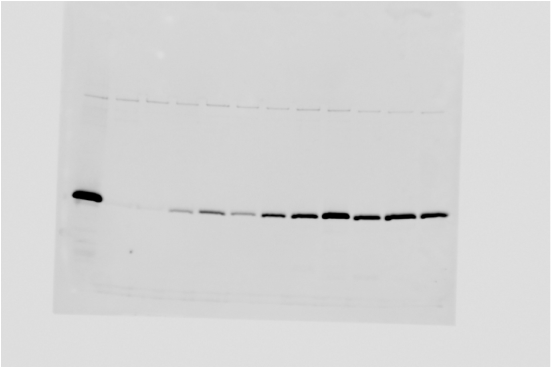

f

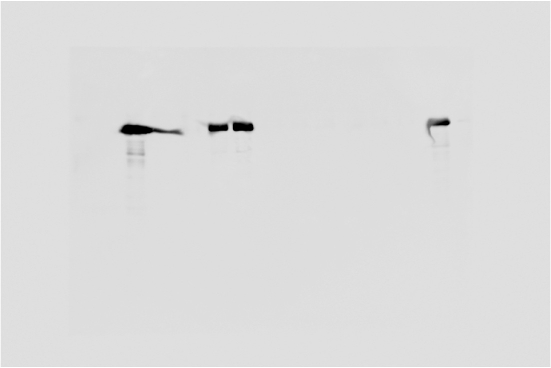

Supplementary Figure 8: Uncropped images used in Figure 5. Labels on each images are corresponding to the panels in Figure 5c–f.

Supplementary Table 1: Strains and plasmids used in this study.

Strains or Plasmids	Genotype or Description	Reference or Source
Strains		
Salmonella enterica serovar Typhimurium		
SJW1103	wild-type for motility and chemotaxis	2
SJW156	*flgD* (frame shift at 72 with 1 additional residue)	3
E. coli		
DH5α	recipient for cloning	NIPPON GENE
BL21(DE3)	*T7* expression host	Novagen
BL21(DE3)pLysS	*T7* expression host, Cm^r	Novagen
B834(DE3)pLysS	*T7* expression host, Cm^r	Novagen
Plasmids		
pET3c	*T7* expression vector, Ap^r	Novagen
pET14b	*T7* expression vector, Ap^r	Novagen
pUC19	multi-copy vector for cloning, Ap^r	NEW ENGLAND BioLabs
pHMK1903	FlgD in pUC19	This study
pHMK2906	N-terminally His-tagged $FlgD_{74\text{-}232}$	This study
pHMK11	modified pTrc99A vector, Ap^r	4
pHMK1901	The *Nde*I-*Bam*HI fragment of pHMK1903 in pUC19	This study
pHMK1921	FlgD (Δ2-10) in pHMK11	This study
pHMK1922	FlgD (Δ11-20) in pHMK11	This study
pHMK1923	FlgD (Δ21-30) in pHMK11	This study
pHMK1924	FlgD (Δ31-40) in pHMK11	This study
pHMK1925	FlgD (Δ41-50) in pHMK11	This study
pHMK1926	FlgD (Δ51-60) in pHMK11	This study
pHMK1927	FlgD (Δ61-70) in pHMK11	This study
pHMK1928	FlgD (Δ71-80) in pHMK11	This study
pHMK1929	FlgD (Δ81-90) in pHMK11	This study
pHMK1930	FlgD (Δ91-100) in pHMK11	This study
pHMK1931	FlgD (Δ2-10) in pET3c	This study
pHMK1932	FlgD (Δ11-20) in pET3c	This study
pHMK1933	FlgD (Δ21-30) in pET3c	This study
pHMK1934	FlgD (Δ31-40) in pET3c	This study
pHMK1935	FlgD (Δ41-50) in pET3c	This study
pHMK1936	FlgD (Δ51-60) in pET3c	This study
pHMK1937	FlgD (Δ61-70) in pET3c	This study
pHMK1938	FlgD (Δ71-80) in pET3c	This study
pHMK1939	FlgD (Δ81-90) in pET3c	This study
pHMK1940	FlgD (Δ91-100) in pET3c	This study

Cm; chloramphenicol, Ap; ampicillin.

Supplementary Table 2: A summary of the data collection statistics for *S. enterica* FlgD crystals.

		SeMet-derivative		
	Native	Peak	Inflection	Remote
Space group	$P3_221$		$P3_221$	
Unit-cell parameters (Å)	*a* = *b* = 141.28, *c* = 153.49		*a* = *b* = 141.22, *c* = 152.71	
Wavelength (Å)	1.0000	0.97910	0.97937	0.99509
Resolution range (Å)	47.84–3.30 (3.48–3.30)	44.1–3.60 (3.79–3.60)	44.1–3.60 (3.79–3.60)	44.1–3.60 (3.79–3.60)
Observed reflections	387528 (57876)	107896 (15822)	108189 (15954)	102983 (13638)
Unique reflections	27142 (3930)	20738 (2974)	20817 (2995)	20807 (2980)
Completeness (%)	100 (100)	99.6 (99.7)	99.5 (99.6)	98.7 (98.0)
Redundancy	14.3 (14.7)	5.2 (5.3)	5.2 (5.3)	4.9 (4.6)
Mean(I)/sd(I)	19.0 (4.3)	14.5 (6.0)	11.5 (4.7)	9.2 (3.3)
R_{merge} (%)	10.9 (40.1)	6.5 (21.5)	8.2 (26.5)	10.0 (35.7)
R_{ano} (%)		4.3 (11.6)	4.1 (13.4)	4.6 (20.6)

Statistics for the highest-resolution shell are shown in parentheses.

Supplementary Table 3: A summary of the data collection statistics for *S. enterica* $FlgD_{74-232}$ crystals.

	Native	Os-derivative
		Peak
Space group	$P2_12_12$	$P2_12_12$
Unit-cell parameters (Å)	*a* = 75.99,	*a* = 76.29,
	b = 104.32,	*b* = 105.15,
	c = 43.87	*c* = 44.28
Wavelength (Å)	1.0000	1.13981
Resolution range (Å)	35.7–2.20	44.28–2.50
	(2.320–2.20)	(2.64–2.50)
Observed reflections	130768 (18531)	184701 (26794)
Unique reflections	18336 (2615)	12733 (1808)
Completeness (%)	99.8 (99.6)	99.1 (98.7)
Redundancy	7.1 (7.1)	14.5 (14.8)
Mean(*I*)/sd(*I*)	14.6 (5.9)	23.2 (4.6)
R_{merge} (%)	8.1 (27.2)	6.4 (56.1)
R_{ano} (%)	-	2.7 (15.1)

Statistics for the highest-resolution shell are shown in parentheses.

Supplementary Table 4: A summary of polar contacts found between subunits in the hook tip.

Subunit/Residues	Atoms	Subunit/Residues	Atoms	Distance (Å)
A		B		
Gln84	$N^{\varepsilon 2}$	Asp86	$O^{\delta 1}$	2.8
Gln84	$N^{\varepsilon 2}$	Ser82	O	3.2
Gln84	O	Gln89	$N^{\varepsilon 2}$	3.1
Ser91	O^{γ}	Gln92	$N^{\varepsilon 2}$	2.8
Val206	N	Ile231	O	3.1
Val206	O	Ile231	N	2.8
Thr207	$O^{\gamma 1}$	Gln230	$N^{\varepsilon 2}$	2.5
Lys208	N	Arg229	O	2.7
Lys208	N^{ζ}	Asp226	O	3.3
A		E		
Gln92	$N^{\varepsilon 2}$	Ser88	O	3.4
Arg229	O	Lys208	N	2.9
Gln230	$N^{\varepsilon 2}$	Thr207	$O^{\gamma 1}$	2.7
Gln231	N	V206	O	2.7
Gln231	O	V206	N	2.9

Supplementary References

1. Pei, J., Kim, B.H. & Grishin, N.V. PROMALS3D: a tool for multiple protein sequence and structure alignments. *Nucleic Acids Res* **36**, 2295-300 (2008).

2. Yamaguchi, S., Fujita, H., Sugata, K., Taira, T. & Iino, T. Genetic analysis of H2, the structural gene for phase-2 flagellin in *Salmonella*. *J Gen Microbiol* **130**, 255-65 (1984).

3. Ohnishi, K., Ohto, Y., Aizawa, S., Macnab, R.M. & Iino, T. FlgD is a scaffolding protein needed for flagellar hook assembly in *Salmonella typhimurium*. *J Bacteriol* **176**, 2272-81 (1994).

4. Kojima, S., Furukawa, Y., Matsunami, H., Minamino, T. & Namba, K. Characterization of the periplasmic domain of MotB and implications for its role in the stator assembly of the bacterial flagellar motor. *J Bacteriol* **190**, 3314-22 (2008).
