# Supplementary material for: Structure of the bacterial flagellar hook cap provides insights into a hook assembly mechanism
Source: Commun Biol. 2021 Nov 16;4:1291. doi: 10.1038/s42003-021-02796-6 (PMC8595650; doi:10.1038/s42003-021-02796-6)
Supplement: Supplementary file 3 — Description of Additional Supplementary Files [file 42003_2021_2796_MOESM3_ESM.pdf]

Description of Additional Supplementary Files

File name: Supplementary Movie 1

Description: Video showing the shape of the pentamer of FlgD
